# Cryoglobulinaemic Vasculitis Secondary to Parvovirus B19 Infection: A Case-Based Review

**DOI:** 10.31138/mjr.120525.erl

**Published:** 2025-12-31

**Authors:** Fernando Albuquerque, Sara Costa, Marcelo Neto, Filipa Canhão André, Maria João Salvador

**Affiliations:** Serviço de Reumatologia da Unidade Local de Saúde de Coimbra, Portugal

**Keywords:** arthritis, cryoglobulinemia, parvovirus b19, human, purpura, vasculitis

## Abstract

Parvovirus B19 (B19V) is a common viral agent that may cause arthritis in adults. In rare cases, B19V has been associated with cryoglobulinaemic vasculitis. We report the case of a 47-year-old woman who presented with an acute febrile polyarthritis and palpable purpura on the lower limbs. Laboratory investigations revealed low complement C4, positive IgM and IgG for Parvovirus B19, and detectable serum cryoglobulins, which immunofixation was consistent with type III cryoglobulinemia. The patient responded favourably to corticosteroid therapy, with complete resolution of symptoms. One month after premature discontinuation of steroids, she experienced a relapse of polyarthralgia. A second course of low-dose corticosteroids led to sustained remission, without further relapse. In order to understand the clinical features of B19V-associated cryoglobulinaemic vasculitis, we conducted a narrative review of the literature using the PubMed database, in which we identified seven cases. The most frequent features included constitutional symptoms, cutaneous vasculitis, joint involvement and complement consumption. One previous case clearly identified type III cryoglobulinemia. These findings highlight the clinical heterogeneity of this entity and the importance of considering B19V in the differential diagnosis of small-vessel vasculitis with cryoglobulinemia, particularly in seronegative patients for hepatitis C and autoimmune diseases.

## INTRODUCTION

Human parvovirus B19 (B19V) is a small, non-enveloped DNA virus primarily known for causing erythema infectiosum in children, characterised by a distinctive facial rash and mild systemic symptoms.^1^ In adults, B19V infection often presents differently, with arthralgia and arthritis being the predominant symptoms, particularly in women. These joint manifestations are typically symmetric and involve small joints, resembling rheumatoid arthritis, but are usually self-limiting.^2^ Beyond these common presentations, B19V can occasionally lead to more severe complications, including haematological disorders, myocarditis, and vasculitis.^1^ Cryoglobulins are circulating immunoglobulins that precipitate at temperatures below core body temperature and redissolve upon rewarming.^3^ When these cryoglobulins deposit in small to medium-sized blood vessels, they can trigger an immune complex-mediated vasculitis, known as cryoglobulinaemic vasculitis. While hepatitis C virus is the most common infectious cause of cryoglobulinaemic vasculitis, other viruses, including hepatitis B and human immunodeficiency virus, have also been implicated. The pathogenesis involves chronic immune stimulation leading to the production of cryoglobulins, which can cause a range of clinical manifestations from purpura to glomerulonephritis.^4^

Although rare, there is growing evidence linking B19V infection to the development of mixed cryoglobulinaemia and subsequent vasculitis. Some case reports have documented patients presenting with vasculitic symptoms and detectable cryoglobulins following acute B19V infection, suggesting a potential causal relationship.^5,6^ This association underscores the need for heightened clinical awareness, especially in patients presenting with vasculitic symptoms and recent viral illness. In this context, we present a case of type III cryoglobulinaemic vasculitis secondary to B19V infection, accompanied by a narrative review of the literature to explore this uncommon but significant clinical entity.

## CASE DESCRIPTION

We report the case of a 47-year-old female patient who presented to the emergency department with a one-week history of fever with a maximum temperature of 38.5 °C, fatigue, diffuse myalgia affecting both upper and lower limbs, and inflammatory arthralgia involving the wrists, knees, and ankles, accompanied by cutaneous lesions on the lower limbs. Her past medical history included allergic rhinosinusitis, supraventricular tachycardia, and chronic gastritis. Chronic medications included bisoprolol 5 mg/day and pantoprazole 20 mg/day.

**Figure 1.**
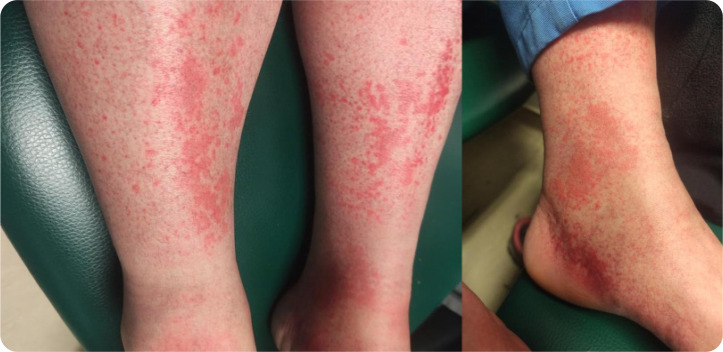
Bilateral palpable purpuric maculopapular lesions on the lower limbs, consistent with cutaneous vasculitis.

On physical examination, there were signs of arthritis in the wrists and ankles, characterised by joint swelling, tenderness and limitation of both active and passive range of motion. Dermatological assessment revealed bilateral palpable purpuric maculopapular lesions on the lower limbs, consistent with cutaneous vasculitis (**Figure 1**).

Initial laboratory workup revealed a mild elevation of erythrocyte sedimentation rate (ESR) of 24 mm/h (normal range: 1 – 20 mm/h), urinary sediment with 14 erythrocytes per high-power field (normal range < 1/high-power field) and dysmorphic erythrocytes, low complement C4 level (0.09 g/L; normal range: 0.15 – 0.57 g/L) (**Table 1**). Complete blood count, serum biochemistry and C-reactive protein (CRP) were normal. Serologies for B and C hepatitis and human immuno-deficiency virus (HIV) were negative. Serum protein electrophoresis was normal, so immunofixation was not performed.

**Table 1.** Laboratory findings at baseline and follow-up.

	At baseline	Time 1	Time 2	Time 3	Reference Range
Leucocytes	6.9	**11.6**	8.3	8.8	3.9 – 10.2 G/L
Hemoglobulin	12.2	11.6	12.5	12.3	12.0 – 16.0 g/dL
Platelets	164	305	227	201	150 – 400 G/L
Creatinine	0.92	1.00	0.81	0.96	0.55 – 1.02 mg/dL
Urinalysis	**14 erythrocytes/HPF Erythrocytes dysmorphism**	Normal	Normal	Normal	-
Urinary Protein/Creatinine Ratio	-	42	66	33	< 200 mg/g
CRP	0.40	0.21	0.23	0.16	< 0.50 mg/dL
ESR	**24**	-	14		1 – 20 mm/h
ANCA	Negative	-	-	-	-
ANA	Negative	-	-	-	-
RF	< 9	-	-	-	< 20 IU/mL
ACPA	0.8	-	-	-	< 7 U/mL
C3	0.96	-	1.23	1.14	0.83 – 1.93 g/L
C4	**0.09**	-	0.18	0.16	0.15 – 0.57 g/L
Parvovirus B19 (IgM)	-	**36.9**	**46.2**	0.7	< 0.9 UI/mL
Parvovirus B19 (IgG)	-	**15**	**64**	**100**	< 2.0 UI/mL
Cryoglobulins	-	**Polyclonal IgM and IgG**	-	Negative	-
HBV	Negative	-	-	-	-
HCV	Negative	-	-	-	-
HIV	Negative	-	-	-	-

Time 1 corresponds to the first follow-up visit, performed one week after initial assessment. Time 2 refers to the second follow-up visit, conducted one month after Time 1. Time 3 represents the third follow-up visit, two months after Time 2. ACPA: anti-citrullinated protein antibodies; ANA: Antinuclear antibodies; ANCA: Antineutrophil cytoplasmic antibodies; CRP: C-reactive protein; ESR: Erythrocyte sedimentation rate; HBV: Hepatitis B virus; HCV: Hepatitis C virus; HIV: Human immunodeficiency virus; HPF: High power field; Ig: Immunoglobulin; RF: Rheumatoid factor.

The patient was started on oral prednisolone 20 mg/day, given the presence of a cutaneous small vessel vasculitis and acute febrile polyarthritis, without evidence for bacterial infection. At that time, the Nephrology team considered the likelihood of renal involvement to be low.

One week later, in the rheumatology outpatient clinic, the patient reported a complete resolution of systemic symptoms and cutaneous lesions. On physical examination, there were no signs of arthritis or vasculitic rash. At this time, further laboratory evaluation showed normalisation of ESR and urinary findings. Antinuclear antibodies (ANAs), antineutrophil cytoplasmic antibodies (ANCAs), rheumatoid factor (RF) and anti-citrullinated protein antibodies (ACPA) were negative. However, serologies for B19V were positive for both IgM and IgG, detected by chemiluminescence immunoassay (CLIA), consistent with B19V acute infection, and serum cryoglobulins were detected. Immunofixation identified polyclonal IgG and IgM, consistent with type III mixed cryoglobulinemia (**Table 1**).

Taken together, the clinical presentation, laboratorial findings, and favourable response to corticosteroids supported a diagnosis of B19V-associated type III cryoglobulinaemic vasculitis.

A gradual tapering of prednisolone was proposed, but the patient discontinued prednisolone on her own initiative. One month later, she reported a worsening of her condition, with inflammatory arthralgia affecting her shoulders, wrists, knees and ankles, with minimal improvement with non-steroidal anti-inflammatory drugs (NSAID). She denied other systemic symptoms. On physical examination, there was joint tenderness in the wrists and ankles, but no swelling was detected, and there were no mucocutaneous lesions or signs of systemic vasculitis. Laboratorial assessment revealed normal blood count and acute phase reactants, but B19V serology remained compatible with ongoing infection (**Table 1**). Since there was no improvement with NSAIDs, regarding the prior manifestations of the disease, we opted to treat with a low dose of prednisolone (5 mg/day).

At a follow-up visit two months later, the patient reported complete resolution of symptoms, although she had voluntarily discontinued corticosteroids one week earlier without medical advice. At this point, serologic testing indicated past B19V infection and cryoglobulins were no longer detectable. The patient remained asymptomatic at 12 months of follow-up, with no clinical or laboratory evidence of disease relapse.

## DISCUSSION

This case illustrates a rare clinical manifestation of B19V infection in a previously healthy adult woman who presented with acute febrile polyarthritis, a presentation characteristic of viral arthritis. However, the concurrent presence of palpable purpuric lesions on the lower limbs, consistent with cutaneous small-vessel vasculitis, raised suspicion for an immune-mediated process beyond typical viral arthritis. Inflammatory markers were largely unremarkable, with only a mild elevation in ESR. The presence of erythrocyturia with dysmorphic erythrocytes initially raised concern for possible glomerular involvement, although nephrology evaluation considered renal involvement unlikely and recommended follow-up urinalysis. In the absence of clinical or laboratory signs of bacterial infection and severe organ involvement or life-threatening features, empirical treatment with medium dose of corticosteroid was initiated, resulting in complete resolution of symptoms within one week. However, the patient stopped the corticosteroid without medical guidance and shortly thereafter the inflammatory polyarthralgia relapsed, without cutaneous or systemic signs of vasculitis. Since there was evidence of ongoing infection and minimal relief with NSAIDs, we opted to initiate treatment with a low dose of corticosteroid which resulted in complete resolution of her symptoms. After discontinuation of corticosteroids, the patient remained asymptomatic.

It should be noted that the patient did not fulfil the classification criteria for cryoglobulinaemic vasculitis,^7^ due to the absence of a second confirmatory cryoglobulin test. Nonetheless, we considered that the clinical context, including the presence of cutaneous vasculitis, arthritis, constitutional symptoms, C4 consumption, cryoglobulinemia, and evidence of B19V infection, supported this diagnosis.

Given the rarity of this presentation and the limited number of reports available in the literature, we performed a narrative literature review following the CABARET recommendations to explore and contextualise other published cases of cryoglobulinaemic vasculitis associated with B19V infection in adults, using the MEDLINE/PubMed and the Directory of Open Access Journals (DOAJ) database up to april 2025. In MED-LINE/PubMed, we used the query “Parvovirus B19” AND (“Cryoglobulinemia” OR “cryoglobulinemic vasculitis”). For the DOAJ database, we conducted two separate queries: “Parvovirus B19” AND “Cryoglobulinemia” and “Parvovirus B19” AND “cryoglobulinemic vasculitis”. We included case reports and case series involving adult patients (≥18 years) with cryoglobulinaemic vasculitis attributed to B19V infection. Studies on paediatric populations, cases secondary to other aetiologies, or without original clinical descriptions were excluded. This strategy identified 26 records, from which five articles were included after screening. These comprised case reports describing adult patients diagnosed with cryoglobulinaemic vasculitis associated with B19V infection. Additionally, one multicentre observational study was identified manually during manuscript preparation, as it had not been retrieved by the original search strategy but reported three relevant cases. Of these, two were included; the third case had already been published separately in a previous report by Chiche et al. and was therefore excluded to avoid duplication.

Among the seven cases of cryoglobulinaemic vasculitis associated with B19V infection included in this review (summarised in **Table 2**), constitutional symptoms such as fever and fatigue were present in five cases. Cutaneous vasculitis was reported in six patients, manifesting as palpable purpura, necrotic skin lesions, or histologically confirmed leukocytoclastic vasculitis. One of these cases progressed to extensive cutaneous necrosis requiring surgical amputation.^8^ Articular symptoms were observed in four patients, including arthralgia or arthritis. Renal involvement occurred in two cases, both presenting with proteinuria and impaired renal function, one of which required haemodialysis.^9^ Pulmonary involvement was reported in one case with concurrent respiratory distress,^9^ and peripheral nervous system involvement was reported in one patient, presenting as ulnar mononeuritis.^10^

**Table 2.** Summary of reported adult cases of cryoglobulinaemic vasculitis associated with parvovirus B19 infection.

**Study**	**Age/Sex**	**Cryoglobulinemia Type**	**Clinical manifestations**	**Complement consumption**	**Rheumatoid Factor**	**Parvovirus serology**	**Repeated cryoglobulins**
Chiche et al.^5^	37/F	Type II	Fever, polyarthritis, myalgia	Low C4	Elevated	IgM and IgG +	Yes (positive after six months of follow-up, then negative)
Cherif et al.^8^	42/F	Mixed (unspecified)	Purpura, fever	Not reported	Not reported	IgM + IgG –	Not reported
Lazzerini et al.^6^	68/M	Type II	Fever, fatigue, polyarthralgia, purpura, acute kidney injury	Low C3 and C4	Elevated	IgM and IgG +	Not specified
Gorse et al.^9^	62/M	Type II	Fever, polyarthralgia, purpura, anuria and pulmonary distress	Low C3, C4 and CH50	Elevated	IgM and IgG +	Yes (negative after treatment)
Kechaou et al.^11^	47/F	Mixed (unspecified)	Fever, Pseudo- erysipelas	Low C3 and C4	Not reported	IgM + IgG –	Not reported
Marion et al.^10^	42/F	Type III	Left ulnar mononeuritis, necrotic purpura	Not reported	Elevated	IgM and IgG +	Not reported
Marion et al.^10^	39/M	Type II	Palpable purpura, subcutaneous nodules, arthralgia	Not reported	Not reported	IgM and IgG +	Not reported

F: Female; M: Male.

Laboratory data revealed elevated acute phase reactants (CRP or ESR) in six patients. Complement consumption, evidenced by low C3 and/or C4 levels, was documented in four cases. Rheumatoid factor was elevated in four cases, although it was not reported in the other three cases. A type II cryoglobulinemia was identified in four patients and a type III in one, while the remaining two had mixed cryoglobulinemia not further specified. All seven patients had serologic evidence of acute B19V infection, and in three cases, viral DNA was confirmed by PCR in the serum or cryoprecipitate, supporting a role for active viral replication in disease pathogenesis.

Repetition of cryoglobulin testing was explicitly reported in two cases. In the case described by Gorse et al.,^9^ cryoglobulins became undetectable during follow-up, coinciding with clinical remission and viral clearance. In contrast, Chiche et al.^5^ reported persistent cryoglobulinemia and anti-B19 IgM antibodies for up to six months, despite spontaneous clinical recovery. These contrasting findings highlight the dynamic and heterogeneous course of B19V-associated cryoglobulinemia and reinforce the importance of serial testing when monitoring disease resolution.

Taken together, these findings highlight the diagnostic value of a careful clinical assessment and temporal correlation with viral serology in suspected cases of cryoglobulinaemic vasculitis, especially when formal classification criteria are not fully met. They also support the hypothesis that B19V may induce immune complex-mediated vasculitis, including cryoglobulinemia, particularly in seronegative patients for hepatitis C or autoimmune diseases.

However, we acknowledge several limitations of our report. First, the diagnosis of cryoglobulinaemic vasculitis was not confirmed by a second positive cryoglobulin test, which may affect diagnostic certainty. Second, the absence of histological confirmation of vasculitis through skin biopsy limits pathological validation, although the clinical features were strongly suggestive. Lastly, as with any narrative review, the selection of cases may be influenced by reporting bias and incomplete data in published reports, potentially underestimating the true frequency or spectrum of B19V-associated cryoglobulinemia.

To our knowledge, this is the first report published in the Mediterranean Journal of Rheumatology highlighting Parvovirus B19-associated type III cryoglobulinaemic vasculitis, emphasising the clinical relevance of this case.

## CONCLUSION

This report reinforces the emerging recognition of B19V as an uncommon but important etiological factor in adult-onset cryoglobulinaemic vasculitis. The presence of constitutional symptoms, cutaneous vasculitis, arthritis, complement consumption, and cryoglobulinemia in temporal association with acute B19V infection supports this diagnosis, even in the absence of a second confirmatory cryoglobulin test. The cases identified in the literature review underscore the clinical heterogeneity of cryoglobulinaemic vasculitis secondary to B19V infection, with presentations ranging from mild, self-limiting disease to severe, organ-threatening manifestations. Notably, only one previous case was clearly identified as a type III cryoglobulinemia, highlighting the diagnostic and scientific relevance of the present report. This case emphasises the importance of considering B19V in the etiological work-up of small-vessel vasculitis with cryoglobulinemia, particularly when classical causes such as hepatitis C are excluded.
